# Defining the Contribution of CYP1A1 and CYP1A2 to Drug Metabolism Using Humanized CYP1A1/1A2 and Cyp1a1/Cyp1a2 Knockout Mice[Fn FN4]

**DOI:** 10.1124/dmd.119.087718

**Published:** 2019-07-19

**Authors:** Y. Kapelyukh, C. J. Henderson, N. Scheer, A. Rode, C. R. Wolf

**Affiliations:** Systems Medicine, School of Medicine, University of Dundee, Jacqui Wood Cancer Centre, Ninewells Hospital, Dundee, United Kingdom (Y.K., C.J.H., C.R.W.) and Taconic Biosciences Inc., Rensselaer, New York (N.S., A.R.)

## Abstract

Cytochrome P450s CYP1A1 and CYP1A2 can metabolize a broad range of foreign compounds and drugs. However, these enzymes have significantly overlapping substrate specificities. To establish their relative contribution to drug metabolism in vivo, we used a combination of mice humanized for CYP1A1 and CYP1A2 together with mice nulled at the Cyp1a1 and Cyp1a2 gene loci. CYP1A2 was constitutively expressed in the liver, and both proteins were highly inducible by 2,3,7,8-tetrachlorodibenzodioxin (TCDD) in a number of tissues, including the liver, lung, kidney, and small intestine. Using the differential inhibition of the human enzymes by quinidine, we developed a method to distinguish the relative contribution of CYP1A1 or CYP1A2 in the metabolism of drugs and foreign compounds. Both enzymes made a significant contribution to the hepatic metabolism of the probe compounds 7-methoxy and 7-ehthoxyresorufin in microsomal fractions from animals treated with TCDD. This enzyme kinetic approach allows modeling of the CYP1A1, CYP1A2, and non-CYP1A contribution to the metabolism of any substrate at any substrate, inhibitor, or enzyme concentration and, as a consequence, can be integrated into a physiologically based pharmacokinetics model. The validity of the model can then be tested in humanized mice in vivo.

**SIGNIFICANCE STATEMENT:**

Human CYP1A1 and CYP1A2 are important in defining the efficacy and toxicity/carcinogenicity of drugs and foreign compounds. In light of differences in substrate specificity and sensitivity to inhibitors, it is of central importance to understand their relative role in foreign compound metabolism. To address this issue, we have generated mice humanized or nulled at the Cyp1a gene locus and, through the use of these mouse lines and selective inhibitors, developed an enzyme kinetic-based model to enable more accurate prediction of the fate of new chemicals in humans and which can be validated in vivo using mice humanized for cytochrome P450–mediated metabolism.

## Introduction

CYP1A2 is a major cytochrome P450 (P450) which accounts for ∼12% of the total hepatic P450 content in humans (Iwatsubo et al., 1997; Achour et al., 2014). CYP1A2 substrates include drugs, industrial chemicals, and environmental toxicants. The enzyme activity is variable in humans due to a combination of genetic polymorphism and environmental factors affecting enzyme expression level and activity. The expression of both CYP1A1 and CYP1A2 is highly regulated by the aryl hydrocarbon receptor (AHR). In the case of hepatic CYP1A2, this can be induced up to 10-fold by AHR ligands (Abraham et al., 2002). The activated AHR binds to xenobiotic response elements on the 5′ flanking region of the CYP1A2 gene. As this element is shared between *CYP1A2* and *CYP1A1* genes, many *AHR* and even some constitutive androstane receptor ligands simultaneously induce both enzymes (Corchero et al., 2001; Ueda et al., 2006; Yoshinari et al., 2008, 2010). The active sites of both CYP1A1 and CYP1A2 have a CYP1 family–specific distortion of the F helix in the area of the substrate binding cavity, causing bending of the helix and resulting in the formation of an enclosed and planar substrate binding site. This explains the overlapping substrate and inhibitor specificities for both enzymes (Sansen et al., 2007; Walsh et al., 2013).

For example, CYP1A1 and CYP1A2 both activate many procarcinogens and/or participate in their detoxification (Nebert et al., 2013; Stiborova et al., 2014). In certain cases, they also mediate the elimination/metabolic activation of drugs (Lin et al., 2017). As a consequence, for any studied AHR ligand (potential environmental toxicant or new pharmaceutical chemical entity), the individual contribution of both CYP1A1 and CYP1A2 to compound elimination in humans needs to be estimated using physiologically based pharmacokinetic (PBPK) models (Andersen et al., 1997; Santostefano et al., 1998; Cortessis and Thomas, 2004). Particular attention has to be given to AHR-mediated induction of the enzymes, as this can markedly affect the relative contribution of CYP1A1 and CYP1A2 to metabolism. For example, 7-ethoxyresorufin *O*-deethylation (EROD) in untreated rat liver microsomes is catalyzed by CYP2C6, CYP2B1, CYP2C11, and CYP3A1/2, whereas the same compound is oxidized predominantly by CYP1A1 in microsomes from 3-methylcholantrene–treated rats with CYP1A2 playing a secondary role (Burke et al., 1994). Similarly, whereas CYP1A2 is considered the sole enzyme catalyzing 7-methoxyresorufin *O*-demethylation (MROD) in liver microsomes from untreated rats (Burke et al., 1994; Floreani et al., 2012), experimental data suggest CYP1A1 to be a second major enzyme involved in the reaction in the liver microsomes from safrole- (Burke et al., 1994) or benzo[a]pyrene-treated animals (Floreani et al., 2012).

Recombinant rat CYP1A1 is ∼59 times more active in EROD and ∼14 times less active in MROD compared to CYP1A2 (Namkung et al., 1988), suggesting that 7-ethoxyresorufin (ER) and 7-methoxyresorufin (MR) are selective substrates for rat CYP1A1 and CYP1A2, respectively. However, recombinant human CYP1A1 is only ∼2.8 times more active in EROD and ∼5.8 times less active in MROD compared with human CYP1A2, indicating more extensive overlap in substrate specificity (Liu et al., 2004). The accuracy of estimates of toxicological risk or drug pharmacokinetic data generated in rodents compared to humans is often compromised by the species differences in metabolism (Cheung and Gonzalez, 2008) and, in a number of cases, differences between rodent and human CYP1A1 and CYP1A2 (Turesky et al., 1998; Turteltaub et al., 1999; Shinkyo et al., 2003). One approach to improving the predictive power of animal models involves humanization for the relevant xenobiotic metabolizing enzyme (Cheung and Gonzalez, 2008). Mouse models humanized for CYP1A1 and/or CYP1A2 have demonstrated human-like procarcinogen activation/detoxification patterns for 2-amino-1-methyl-6-phenyl-imidazola[4,5-b]pyridine (Cheung et al., 2005) and aristolochic acid I (Levova et al., 2012).

In this study, two new mouse models are described. In one [human CYP1A1/1A2 (hCYP1A1/1A2)], both mouse *Cyp1a1* and *Cyp1a2* were replaced with the corresponding human orthologs. The second model (Cyp1a KO) is a *Cyp1a1* and *Cyp1a2* knockout. The expression of the Cyp1a/CYP1A enzymes was measured in liver and extrahepatic tissues of vehicle and 2,3,7,8-tetrachlorodibenzo-p-dioxin (TCDD)–treated animals. A method for the assessment of relative contribution of CYP1A1 and CYP1A2 to EROD and MROD was developed using the CYP1A1 selective inhibitor quinidine. The method was applied to measure individual contributions of human CYP1A1 and CYP1A2 to EROD and MROD in liver microsomes from TCDD-treated humanized mice. Values of fraction metabolized by CYP1A2 were determined for the CYP1A2 substrates tacrine, ramelteon, and caffeine in untreated humanized and wild-type (WT) animals. The utility of this mouse line relative to the recently created more-complex P450 humanized model (Henderson et al., 2019) is discussed.

## Materials and Methods

### Generation of hCYP1A1/1A2 and Cyp1a KO Mice

hCYP1A1/1A2 and Cyp1a KO mice were generated in a collaboration between CXR Biosciences (now Concept Life Sciences) and Taconic Biosciences in a project funded by the Scottish Government through the Intermediate Technology Institutes (ITI) program (principal investigators C.R.W. and N.S.) as detailed later. Culture and targeted mutagenesis of embryonic stem cells were carried out as described previously (Behringer et al., 2014). The murine *Cyp1a1* and *Cyp1a2* gene loci were successively modified by homologous recombination in C57BL/6NTac mouse embryonic stem cells with two targeting vectors, such that the genomic sequences between the translational start ATGs and the stop codons of mouse *Cyp1a1* and *Cyp1a2* were replaced with the orthologous genomic sequences of human *CYP1A1* and *CYP1A2*, respectively (Supplemental Fig. 1), thus removing the murine Cyp1a1 and Cyp1a2 genes. Southern blot analysis was used to identify correct double-targeted clones, which were injected into BALB/c blastocysts and transferred into foster mothers as described previously (Behringer et al., 2014). Chimeric mice were bred to a germline flipase (Flpe) deleter strain to remove selectable markers (Supplemental Fig. 1) as described previously (Scheer et al., 2012a). Heterozygous *CYP1A1/1A2* humanized mice were identified by polymerase chain reaction and either crossed with each other to generate homozygous hCYP1A1/1A2 mice or crossed to a deleter strain expressing Cre recombinase to remove the *Cyp1a* gene locus from the germ line (Supplemental Fig. 1). Heterozygous *Cyp1a* knockout offspring were identified by polymerase chain reaction and further crossed to generate homozygous Cyp1a KO mice. The Flpe and Cre deleter strains mentioned earlier were generated in house on a C57BL/6NTac genetic background.

### Animal Accommodation and Husbandry

Animal procedures were performed under license from the UK Home Office [Animal (Scientific Procedures) Act (1986), and 2010/63/EU] and after approval by the Ethical Review Committee, University of Dundee. Homozygous mice for each transgenic line were used for experimental studies. C57BL/6NTac mice were used as WT controls. Mice were kept in open-top cages with ad libitum access to food (RM1; Special Diet Services Ltd., Essex, UK) and drinking water, and were acclimatized for at least 5 days before study commencement. Room temperature was between 19°C and 23°C and relative humidity was 40%–70%, with a 12-hour light/dark cycle (Scheer et al., 2012b).

### TCDD Treatment

Mice were given a single intraperitoneal dose of TCDD (10 *µ*g/kg) or vehicle control (corn oil) and then euthanized 48 hours after the dosing using a rising concentration of CO_2_.

### Tissue Collection

Venous blood was removed by cardiac puncture and dispensed into lithium/heparin-coated tubes. Red blood cells were removed by centrifugation (16.1 krcf for 10 minutes at room temperature), and the supernatant (plasma) was stored at approximately −70°C.

The gall bladder was removed, and then the liver was removed, weighed, and scissor-minced in ice-cold KCl [1.15% (w/v)] for subsequent liver subcellular fractionation. The small intestine and colon were removed and flushed with ice cold phosphate-buffered saline (PBS) containing a protease inhibitor cocktail (Roche Diagnostics, Basel, Switzerland).

The duodenum, jejunum, ileum, and colon sections (approximately 10 cm each) were transferred into separate tubes, flash frozen immediately in liquid nitrogen, and stored at approximately –70°C prior to the preparation of microsomes. The heart, lungs, brain, spleen, testis, and kidneys were removed from each animal, flash frozen in liquid nitrogen, and stored at approximately −70°C prior to microsome preparation.

### Preparation of Microsomes

Microsomal fractions were prepared as previously described (Henderson et al., 2019). In brief, fresh liver samples were homogenized in ice-cold SET(Sucrose-EDTA-Tris) buffer (0.25 M sucrose, 5 mM EDTA, 20 mM Tris-HCL, pH 7.4; 9 ml buffer/g liver) and centrifuged at 2000 rpm (Sorvall RTH-250, 10 minutes, 4°C). The supernatant was centrifuged (12,000 rpm, Sorvall SS-34 rotor, 20 minutes, 4°C), the resulting supernatant was centrifuged (∼30,000 rpm, Sorvall TFT-45.6, 90 minutes, 4°C) (ThermoFisher Scientific, UK), and microsomal pellets were resuspended in ice-cold SET buffer and stored at −70°C.

Frozen individual duodenum, ileum, jejunum, and colon samples and pooled (one pool per experimental group) lung, kidney, spleen, heart, brain, and testis were homogenized in SET buffer containing protease cocktail inhibitor (Roche) and PMSF (phenylmethylsulfonyl fluoride) (1 mM) using a Polytron homogenizer. Microsomal fractions were prepared as described earlier for liver tissue.

### Biochemical Measurements

#### Clinical Chemistry.

The activity of alanine aminotransferase and concentration of albumin in the plasma samples were measured in the Clinical Pathology Service Laboratory (Mary Lyon Centre, Harwell, UK).

#### Total Protein and P450 Determination.

Microsomal protein concentration fractions were measured using a modification of a previously described method (Lowry et al., 1951) and total hepatic microsomal P450 as previously described (Omura and Sato, 1964).

### Immunoblotting

The expression of Cyp1a, CYP1A1, and CYP1A2 in pooled liver, duodenum, ileum, jejunum, colon, lung, kidney, spleen, heart, brain, and testis microsomal samples was determined by immunoblot analysis using primary antibodies against recombinant rat CYP1A2 (Forrester et al., 1992), human CYP1A1, and CYP1A2 [AB1258 (Millipore, UK) and PAP 021 (Cypex/Nosan, UK)], loading 20 *µ*g of microsomal protein per lane. The positive standards were membrane preparations from bacteria expressing recombinant human CYP1A1 (0.36 pmol) or CYP1A2 (1 pmol). Protein expression was visualized using Immobilon Western chemiluminescent detection (Millipore) according to the manufacturer’s instructions, and data were collected and processed (contrast/brightness adjusted identically for each across the entire image) using a FujiFilm LAS-3000 mini CCD system and the device software version 2.2. Acquired images were saved in the tagged image file format using MultiGauge software (FujiFilm) and transferred to PowerPoint using Picture Manager (Microsoft Office 2010).

### 7-Ethoxyresorufin *O*-Deethylation and 7-Methoxyresorufin *O*-Demethylation

A mixture of ER (0.93 *μ*M) or MR (0.46 *μ*M) and liver microsomes (0.004–0.27 mg protein/ml) in 100 mM potassium phosphate buffer (pH 7.4) supplemented with MgCl_2_ (3.3 mM) was incubated at 37°C for 5 minutes before the reaction was initiated by injection of NADPH (final concentration, 1.2 mM). Generation of the fluorescent product was registered in a kinetic mode using Fluoroscan Ascent FL (excitation filter, 530 nm; emission filter, 584 nm; Labsystems). Slopes of the linear part of the kinetic curves were calculated using Ascent Software version 2.4.1 (Labsystems). For each well with the reaction medium, there was a control well containing the reaction mixture with resorufin (4 pmol). Before addition of NADPH to the reaction wells, fluorescence was recorded from both the reaction and the control wells. The average fluorescence was calculated, and the difference between wells with and without resorufin was used for the conversion of the relative fluorescence units to the picomoles of the reaction product. Activities in duodenum, ileum, jejunum, colon, lung, kidney, spleen, heart, brain, and testis microsomes were measured as described earlier except for the following adjustments: MR concentration was 1 *μ*M, concentration of total microsomal protein in the reaction was in the range of 0.07–0.43 mg/ml, and amount of resorufin added to calculate the reaction product concentration was 40 pmol.

### Inhibition of Recombinant CYP1A1 and CYP1A2 by Quinidine

A mixture of MR (0.57 *μ*M) and microsomes [3.46 pmol/ml CYP1A1 or 10.8 pmol/ml CYP1A2; 0.25 mg protein/ml adjusted using Control Bactosomes (Cypex)] in phosphate buffer (100 mM KH_2_PO_4_, pH 7.4, 3.3 mM MgCl_2_) was incubated with quinidine (0–2 mM) in a microtiter plate reader for 5 minutes at 37°C prior to the start of the reaction by addition of NADPH (final concentration, 1.2 mM). Generation of fluorescent product was registered in a kinetic mode (excitation filter, 530 nm; emission filter, 584 nm). Slopes of the linear part of the kinetic curves were calculated using Ascent Software version 2.4.1 (Labsystems).

### Estimation of Individual Contribution of CYP1A1 and CYP1A2 to EROD and MROD in Liver Microsomes from hCYP1A1/1A2 Mice

A mixture of ER or MR (0.039–5 *μ*M) and microsomes in phosphate buffer (100 mM KH_2_PO_4_, pH 7.4, 3.3 mM MgCl_2_) was incubated with quinidine [0, 30, and 200 *μ*M (except for MROD catalyzed by recombinant CYP1A1, where quinidine was at 0, 6, and 30 *μ*M)] in a microtiter plate reader for 5 minutes at 37°C prior to the start of the reaction by addition of NADPH (final concentration, 1.2 mM). Protein enzyme/concentrations were 3.46 pmol/ml for recombinant human CYP1A1, 10.8 pmol/ml for recombinant human CYP1A2, 0.05 mg protein/ml for liver microsomes from TCDD-treated hCYP1A1/1A2 mice, and 0.25 mg protein/ml for liver microsomes from TCDD-treated Cyp1 KO mice and vehicle-treated hCYP1A1/1A2 mice. Where needed, total protein concentration in the reaction mixtures was adjusted using Control Bactosomes (Cypex), so all reactions (including those with the recombinant cytochrome P450s) were carried out at a total protein concentration of 0.25 mg protein/ml. Generation of fluorescent product was registered in a kinetic mode (excitation filter, 530 nm; emission filter, 584 nm). Slopes of the linear part of the kinetic curves were calculated using Ascent Software version 2.4.1 (Labsystems). For each well with the reaction medium, there was a control well containing the reaction mixture with resorufin (40 pmol). Before addition of NADPH to the reaction wells, fluorescence was recorded both from the reaction and from the control wells. The average fluorescence was calculated, and the difference between wells with and without resorufin was used for the conversion of the relative fluorescence units to the picomoles of the reaction product. The selected quinidine concentrations provided marked CYP1A1 inhibition, which is essential for precise calculation of inhibition constants (Kakkar et al., 2000) while leaving sufficient enzyme activity for accurate measurement. Kinetic parameters of quinidine inhibition of recombinant CYP1A1 are presented in Table 1.

**TABLE 1:** Kinetic parameters of EROD and MROD inhibition by quinidine in microsomes

Microsomes Origin	K_s1_ ± S.E.	K_s2_ ± S.E.	K_i_ ± S.E.	*α*	Quinidine Inhibition Type
	*μM*	*μM*	*μM*		
EROD					
rCYP1A1	0.09 ± 0.008	11 ± 1	3.3 ± 0.35	31 ± 8	Mixed
rCYP1A2	(Km) 1.2 ± 0.2	NA	NA	NA	NA
Cyp1a KO	0.83 ± 0.04	13 ± 1.5	422 ± 18	NA	Noncompetitive
MROD					
rCYP1A1	0.47 ± 0.07	1.3 ± 0.18	2.2 ± 0.18	NA	Competitive
rCYP1A2	0.58 ± 0.08	12 ± 3.5	NA	NA	NA
Cyp1a KO	0.96 ± 0.18	3.4 ± 0.8	272 ± 28	NA	Noncompetitive

K_i_, dissociation constant of the enzyme-inhibitor complex; K_s1_, dissociation constant of the productive enzyme-substrate complex; K_s2_, dissociation constant of the inhibitory enzyme-substrate complex; NA, not applicable; rCYP1A1, recombinant CYP1A1; rCYP1A2, recombinant CYP1A2; *α*, parameter describing the effect of inhibitor binding on the binding of the substrate and vice versa.

### Microsomal Stability of Ramelteon and Tacrine

A 880-*μ*l mixture of ramelteon (1.25 *μ*M) and C57BL6J (WT), Cyp1a KO, hCYP1A1/1A2, or pooled human liver microsomes (0.625 mg protein/ml) in phosphate buffer (100 mM KH_2_PO_4_, pH 7.4, 3.3 mM MgCl_2_) was incubated for 5 minutes at 37°C in a water bath before an 80-*μ*l aliquot was mixed with 100 *μ*l of ice-cold methanol containing tacrine (100 ng/ml) as an internal standard, followed by addition of 20 *μ*l of NADPH solution (6 mM) in the phosphate buffer. The reaction was started by addition of 200 *μ*l of NADPH to the remaining mixture of ramelteon with microsomes, and 100-*μ*l aliquots were taken at 1, 2, 5, 10, 15, 20, and 30 minutes after the reaction start; mixed with an equal volume of ice-cold methanol containing the internal standard; incubated on ice for at least 20 minutes; and centrifuged for 15 minutes at 16,000 rcf and +4°C on Centrifuge 5415 R (Eppendorf, Hamburg, Germany). Control incubations were carried out without the cofactor or microsomes. The concentration of ramelteon in the supernatant was measured by high-performance liquid chromatography–tandem mass spectrometry (MS/MS). Chromatographic separation was performed on a Prodigy Phenyl-3 column (5 *μ*m, 50 × 2.0 mm; Phenomenex) using an injection volume of 10 *μ*l and a run time of 7 minutes. Mobile phase consisted of 0.1% solutions of formic acid in water (solvent A) and acetonitrile (solvent B). For elution, a linear gradient from 20% to 60% of solvent B in 4 minutes was used, followed by 3-minute equilibration at 20% B. The multiple reaction monitoring parameters for ramelteon and tacrine were 260.28, 199.2 (precursor ion) and 204.21, 171.11 (product ion), respectively. The concentrations of ramelteon were calculated from the calibration curve. Tacrine microsomal stability was measured as described for ramelteon except that the final microsomal protein concentration was 1 mg/ml; the reaction aliquots were collected at 5, 10, 20, 30, 40, 50, and 60 minutes after the reaction start; and the reaction was stopped by mixing with 120 *μ*l of 167 mM hydrochloric acid containing 183 ng/ml caffeine as the internal standard. The mobile phase gradient started at 5% B. The multiple reaction monitoring parameters for tacrine and caffeine were 199.2, 195.18 (precursor ion) and 171.11, 138.07 (product ion), respectively.

### Caffeine Pharmacokinetics

Caffeine [5 mg/kg; 10 ml/kg; dissolved in 50 mM sodium citrate buffer (pH 4.7)] was delivered to WT, Cyp1a KO, and hCYP1A1/1A2 mice (four animals per experimental group) by oral administration. A separate experimental group consisted of mice with conditionally deleted hepatic P450 oxidoreductase (Henderson et al., 2003) [hepatic reductase null (HRN) mice]. Whole blood samples from WT and hCYP1A1/1A2 mice were collected at 12, 24, and 40 minutes and 1, 2, 3, 4, 6, and 8 hours after the administration. Whole blood samples from HRN and Cyp1a1/1a2 KO mice were collected at 12, 24, and 40 minutes and 1, 3, 6, 8, 12, and 24 hours postadministration. The collected whole blood samples (10 *μ*l) were mixed with an equal volume of heparin solution in water (15 U/ml), frozen in liquid nitrogen, and stored at −70°C. The concentration of caffeine in whole blood was measured by liquid chromatography–MS/MS. Calibration standards were prepared by spiking whole mouse blood with an appropriate amount of caffeine standard and mixing the whole blood aliquots with an equal volume of heparin solution in water (15 U/ml). The test samples and calibration standards (20 *μ*l) were mixed with 80 *μ*l of perchloric acid (1.5%) containing tacrine (50 ng/ml) as internal standard, vortexed, and centrifuged at 16,100 rcf for 15 minutes. The supernatant was collected, and the centrifugation step was repeated. The supernatant was transferred to a 96-well plate, and caffeine concentration was measured by high-performance liquid chromatography–MS/MS from the calibration curve using the same conditions as those described for tacrine.

Pharmacokinetic parameters were calculated using Phoenix WinNonlin version 6.4 (Certara, St. Louis, MO).

Full details of the data analysis are given in the Supplemental Materials.

## Results

### Generation of hCYP1A1/1A2 and Cyp1a KO Mice.

Homozygous hCYP1A1/1A2 and Cyp1a KO mice appeared normal, could not be distinguished from WT animals, and had normal survival rates. There were only minor differences between the different lines with or without TCDD treatment, as shown in Supplemental Table 1.

### Total Cytochrome P450.

**Fig. 1.**
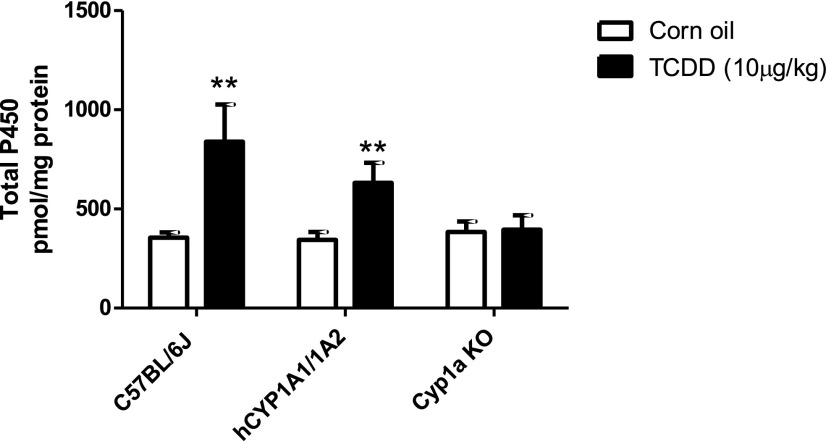
Total cytochrome P450 in liver microsomes from vehicle and TCDD-treated WT, hCYP1A1/1A2, and Cyp1a KO mice. Liver microsomes were prepared from vehicle (corn oil) and TCDD-treated hCYP1A1/1A2, Cyp1a KO, and WT mice (*n* = 4), and total cytochrome P450 content was measured as detailed in *Materials and Methods*. Data are presented as the mean ± S.D.; **significantly different from corresponding corn oil–treated group (unpaired *t* test; two-tailed *P* value; *P* < 0.01).

Total hepatic cytochrome P450 content in untreated WT, hCYP1A1/1A2, and Cyp1a KO mice was similar, suggesting that Cyp1a1 and Cyp1a2 are only minor constitutive P450 forms (Fig. 1). Administration of TCDD resulted in a significant increase of total hepatic cytochrome P450 in WT mice (2.37-fold, from 355 to 840 pmol/mg protein) and hCYP1A1/1A2 (1.84-fold, from 344 to 632 pmol/mg protein). As there was no change in total P450 in Cyp1a KO on TCDD treatment, the increase was attributable to the induction of CYP1A enzymes in WT and hCYP1A1/1A2 mice, which accounted for 58% and 46% of the total hepatic P450 content, respectively.

### CYP1A1 and CYP1A2 Protein Expression and Activity.

**Fig. 2.**
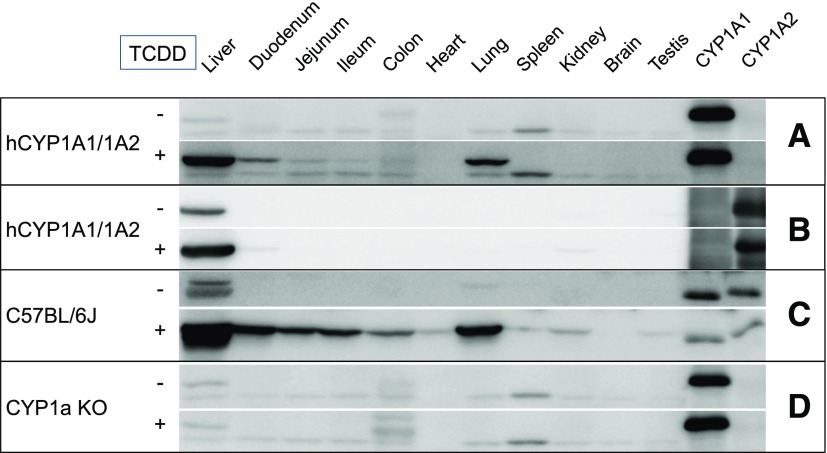
Basal and TCDD-inducible expression of Cyp1a/CYP1A in tissues from WT, Cyp1a KO, and hCYP1A1/1A2 mice. Microsomes were prepared from vehicle (corn oil) and TCDD-treated hCYP1A1/1A2, Cyp1a KO, and WT mice from the tissues shown and immunoblotted for hCYP1A1 (A and D), human CYP1A2 (B), and mouse Cyp1a (C) as detailed in *Materials and Methods*. Standards: CYP1A1 (0.36 pmol/lane) and CYP1A2 (1 pmol/lane) expressed in bacterial membranes.

Western blot analysis showed that CYP1A1 was not expressed constitutively in the humanized mouse liver but CYP1A2 was (Fig. 2, A and B). Neither protein was expressed constitutively in any other tissue. Bands observed in vehicle control samples in Fig. 2A and visible in Cyp1a KO microsomes in Fig. 2D are likely due to nonspecific binding of CYP1A1 antibodies. On TCDD treatment, both CYP1A1 and CYP1A2 were induced in the liver and, in the case of CYP1A1, in the lung and duodenum, with a low level of induction in the ileum and jejunum. The induction appeared to be less than that for Cyp1a1 in WT animals (Fig. 2C), but this may relate to the antibodies used. As expected, both Cyp1a1 and Cyp1a2 (data not shown) could not be detected in the KO animals irrespective of TCDD treatment.

**Fig. 3.**
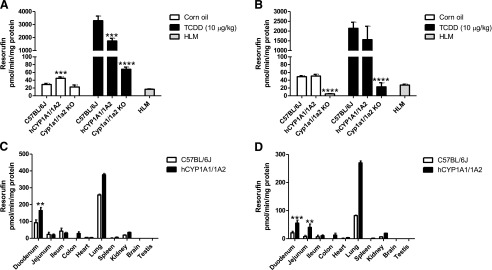
EROD and MROD activities in hepatic and extrahepatic tissues from WT, Cyp1a KO, and hCYP1A1/1A2 mice. Liver microsomes were prepared from vehicle (corn oil) and TCDD-treated hCYP1A1/1A2, Cyp1a KO, and WT mice for the tissues shown, and EROD (A) and MROD (B) activities were measured as detailed in *Materials and Methods*. Microsomes from extrahepatic tissues of TCDD-treated hCYP1A1/1A2 and WT mice were prepared, and EROD (C) and MROD (D) activities were measured. Data are presented as the mean ± S.D. (*n* = 4); *significantly different from corresponding WT group (unpaired *t* test; two-tailed *P* value; **P < 0.01; ***P < 0.001; ****P < 0.0001). Heart, lung, spleen, kidney, brain, and testis microsomes were prepared from pooled organs of each experimental group. Their activities are presented as the mean ± S.D. of three measurements of the pooled sample. No test for statistical significance was performed on these data. HLM, pooled human liver microsomes.

EROD activity in liver microsomes from Cyp1a KO mice was decreased by 22% relative to WT animals, while the constitutive activity was slightly increased (to 155%) in hCYP1A1/1A2 mice (Fig. 3A). Treatment with TCDD resulted in a marked (∼114-fold) increase in EROD activity in WT and 39-fold in hCYP1A1/1A2 mice relative to untreated animals. A 3-fold increase in Cyp1a KO liver was also observed. MROD was decreased by approximately 90% in liver microsomes from Cyp1a KO mice compared with WT animals, whereas in the hCYP1A1/1A2 mouse line the activity was unchanged (Fig. 3B). On TCDD treatment of WT and hCYP1A1/1A2 mice, 44- and 31-fold increases in MROD activities were measured, respectively. A small increase in activity (4.6-fold) was also measured in the Cyp1a KO line.

Both EROD and MROD activities were below the limit of detection in all extrahepatic tissues in all mouse models. On TCDD treatment, these activities were induced in several tissues of both WT and hCYP1A1/1A2 mice. The highest activity for both substrates was in the lungs, with significant activity in the duodenum and other regions of the gastrointestinal tract. These data are consistent with the Western blot analysis of CYP1A1 expression (Fig. 3, A and C). Interestingly, the humanized samples exhibited higher activities than WT samples for these substrates.

### Estimation of the Relative Contribution of CYP1A2 to EROD and MROD Activities.

**Fig. 4.**
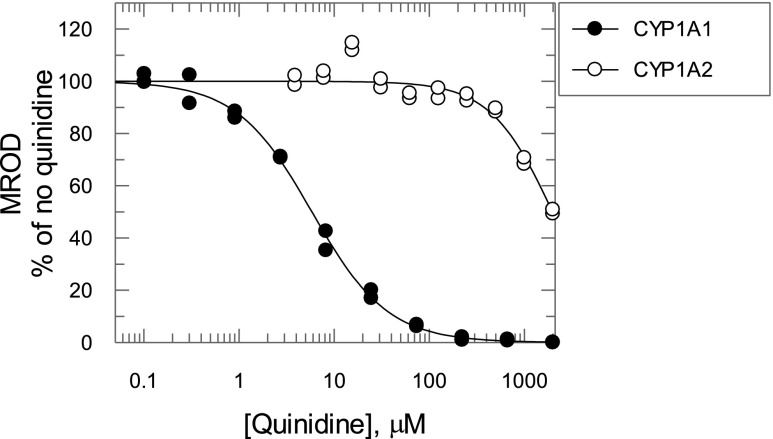
Effect of quinidine on MROD activity catalyzed by recombinant human CYP1A1 and CYP1A2. Recombinant human CYP1A1 (closed circles) and CYP1A2 (open circles) were coexpressed with human P450 reductase in bacterial microsomes, and activity was measured as detailed in *Materials and Methods*.

**Fig. 5.**
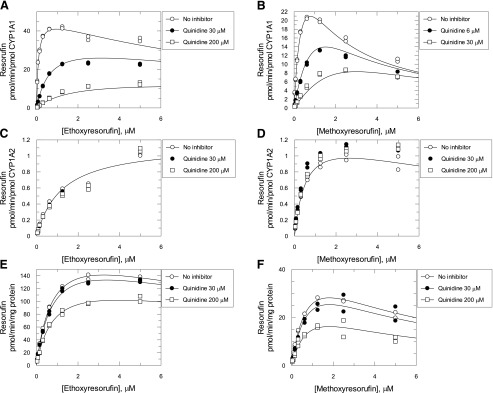
Effect of quinidine on EROD and MROD activities catalyzed by recombinant human CYP1A1, CYP1A2, and liver microsomes from Cyp1a KO mice at different substrate concentrations. EROD (A, C, and E) and MROD (B, D, and F) catalyzed by recombinant human CYP1A1 (A and B), CYP1A2 (C and D), and liver microsomes from TCDD-treated Cyp1a KO mice (E and F) at different concentrations of substrate and quinidine, as detailed in *Materials and Methods*. Symbols are the measured reaction rates. Lines are nonlinear regression analysis of the data using eqs. 1–6 (for details, see *Data Analysis* in the Supplemental Materials).

**Fig. 6.**
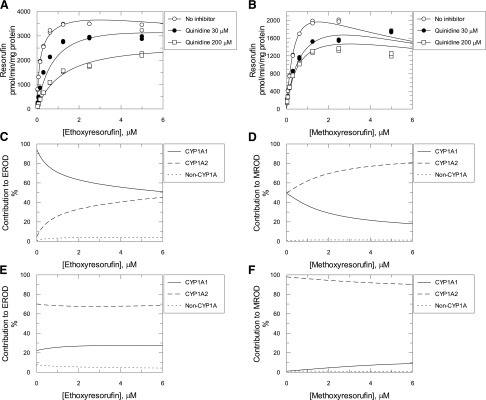
EROD and MROD activities measured in the presence or absence of quinidine in liver microsomes from TCDD-treated hCYP1A1/1A2 humanized mice. EROD (A) and MROD (B) with and without quinidine catalyzed by liver microsomes from TCDD-treated hCYP1A1/1A2 humanized mice. Symbols are the measured reaction rates. Lines are nonlinear regression analysis of the data using eqs. 7 and 8, respectively (for details, see *Data Analysis* in the Supplemental Materials). Contribution of CYP1A1, CYP1A2, and non-CYP1A components of the reactions were calculated using eqs. 9–14 (for details, see *Data Analysis* in the Supplemental Materials) for EROD (C and E) and MROD (D and F) with (E and F) and without (C and D) quinidine (200 *μ*M).

Quinidine is a known inhibitor of the human P450 enzymes CYP2D6 and CYP1A1 but not CYP1A2 (Ching et al., 2001). As CYP2D6 is not involved to any significant extent in either EROD (McGinnity et al., 1999) or MROD (Burke et al., 1994), quinidine was used as a CYP1A1-specific inhibitor in this study. Consistent with the literature (Ching et al., 2001), recombinant CYP1A1–mediated MROD was strongly inhibited by quinidine (Fig. 4, IC_50_ = 5.8 *μ*M), with CYP1A2 having a much lower affinity (1977 *μ*M). We used this difference to determine the relative contribution of CYP1A1, CYP1A2, and cytochrome P450s other than CYP1A to ER and MR metabolism in liver microsomes from TCDD-treated hCYP1A1/1A2 mice. The overall rate of EROD or MROD was assumed to be the addition of rates from the CYP1A1, CYP1A2, and non-Cyp1a components. Initially, the interaction of substrate and quinidine with each one of the aforementioned components was studied individually using human recombinant CYP1A1, CYP1A2, and microsomes from TCDD-treated Cyp1a KO mice. For human CYP1A1, EROD and MROD inhibition by quinidine was consistent with a mixed and competitive mechanism, respectively (Fig. 5, A and B; Supplemental Scheme 1, a and d, eqs. 1 and 4; Table 1). Quinidine did not inhibit either EROD or MROD activity catalyzed by CYP1A2 (Fig. 5, C and D; Supplemental Scheme 1, b and e, eqs. 2 and 5; Table 1). In TCDD-treated Cyp1a KO samples, both EROD and MROD were inhibited noncompetitively with high K_i_ values (>200 *μ*M) (Fig. 5, E and F; Supplemental Scheme 1, c and f, eqs. 3 and 6; Table 1). Substrate and quinidine binding constants were calculated for all of the reactions measured earlier (Table 1) and used in eqs. 7 and 8 to relate EROD and MROD reaction rates to concentrations of substrate and quinidine. It was assumed that the substrate and quinidine binding affinities of recombinant CYP1A1 and CYP1A2 are the same as those of CYP1A1 and CYP1A2 in liver microsomes from TCDD-treated hCYP1A1/1A2 mice. Also, it was assumed that the non-Cyp1a component of both EROD and MROD in hCYP1A1/1A2 mice corresponds to the total EROD and MROD in Cyp1a KO mice. V_max_ values for EROD and MROD of the non-Cyp1a component were those calculated from experiments using liver microsomes from TCDD-treated Cyp1a KO mice. As a result, eqs. 7 and 8 are left with two independent variables—namely, substrate and quinidine concentrations—and two parameters (V_max_ for CYP1A1 and CYP1A2). These V_max_ values were calculated by nonlinear regression analysis of reaction rates in microsomes from TCDD-treated hCYP1A1/1A2 mice at different concentrations of quinidine and ER (Fig. 6A) or MR (Fig. 6B) using eqs. 7 and 8, respectively. The V_max_ values for CYP1A1 and CYP1A2 were 2800 and 1900 pmol/min/mg protein for EROD, and 1600 and 2000 pmol/min/mg protein for MROD, respectively.

With these V_max_ values, all parameters in eqs. 7 and 8 became known, which allowed simulation of the reaction rates in liver microsomes from hCYP1A1/1A2 mice for any given substrate and quinidine concentration not only for the general reaction but also for the individual CYP1A1, CYP1A2, and non-Cyp1a contributions. Using these equations, the contribution of each individual enzymatic component in liver microsomes from TCDD-treated hCYP1A1/1A2 mice for EROD (Supplemental eqs. 9–11; Fig. 6E) and MROD (eqs. 12–14; Fig. 6, D and F) was calculated. In the absence of quinidine, the CYP1A1 contribution to EROD at a low (10 pM) substrate concentration was calculated to be ∼94% (Fig. 6C). This decreased to 70% at 1 *μ*M ER and further reduced to ∼51% at 6 *μ*M substrate. Correspondingly, the contribution of CYP1A2 was increased from ∼4.8% at low substrate concentration to ∼45% at 6 *μ*M ER. The non-Cyp1a contribution was in the range of 0.8%–3.9%. At a quinidine concentration of 200 *μ*M and low concentration of ER, the CYP1A1, CYP1A2, and non-CYP1A contributions were ∼22%, 70%, and 8%, respectively, and did not change substantially with rise of ER concentration.

**Fig. 7.**
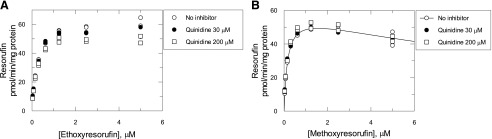
EROD and MROD activities catalyzed by liver microsomes from vehicle-treated hCYP1A1/1A2 humanized mice in the presence or absence of quinidine. EROD (A) and MROD (B) with and without quinidine catalyzed by liver microsomes from vehicle-treated (corn oil) hCYP1A1/1A2 humanized mice. Symbols are the measured reaction rates, and the line is derived from nonlinear regression analysis of the MROD in the absence of quinidine using eq. 5 (for details see *Data Analysis* in the Supplemental Materials).

CYP1A1 and CYP1A2 contributions to MROD activity were almost equal (49% and 50%, respectively) at low substrate concentrations in the absence of inhibitor (Fig. 6D). As MR concentration increased, the CYP1A1 contribution decreased and CYP1A2 contribution increased, being ∼18% and ∼81%, respectively, at 6 *μ*M MR. At low concentrations of MR and 200 *μ*M quinidine, the reaction was almost exclusively catalyzed by CYP1A2 (∼98% contribution) with the CYP1A1 impact being less than 1% (Fig. 6F). As the substrate concentration increased, CYP1A2 contribution decreased to ∼90% and CYP1A1 increased to ∼9%. Non-Cyp1a contribution to MROD did not exceed 1.5%. In liver microsomes from vehicle-treated hCYP1A1/1A2 mice, 200 *μ*M quinidine resulted in minor inhibition of the EROD, which was highest (∼20%) at high substrate concentrations (Fig. 7A). MROD was not affected by the inhibitor (Fig. 7B).

Human recombinant CYP1A1 and CYP1A2 reconstituted from purified enzymes have been reported to have EROD/MROD activity ratios of 6.5 and 0.4, respectively, at 10 *μ*M substrate (Liu et al., 2004). The EROD/MROD ratio for CYP1A1 in hCYP1A1/1A2 liver microsomes was calculated by eqs. 1 and 4 using the following assumptions: 1) 10 *μ*M substrate concentration; 2) absence of inhibitor; 3) substitution of the substrate binding parameters with corresponding calculated values from Table 1; and 4) substitution of V_max_ parameters for 2800 and 1600 pmol/min/mg protein for EROD and MROD, respectively. The calculated EROD/MROD activity ratio using this approach was 8.1 times for CYP1A1, in good agreement with the published data. The same ratio for CYP1A2 calculated by eqs. 2 and 5 and using V_max_ values of 1909 and 1969 pmol/min/mg protein for EROD and MROD, respectively, resulted in an activity ratio of 1.64, suggesting a decreased CYP1A2 preference for MROD in mouse liver microsomes compared with the reconstituted recombinant enzyme system. It should be noted that at 0.5 *μ*M MR, the calculated EROD/MROD activity ratio was 0.63, suggesting a higher CYP1A2 preference for MROD at low substrate concentrations. However, at high substrate concentrations, the ratio changed, possibly due to substrate inhibition.

### Microsomal Stability of Ramelteon and Tacrine.

**Fig. 8.**
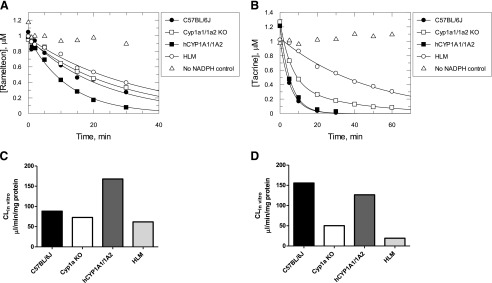
Microsomal stability and in vitro clearance of ramelteon and tacrine. Microsomal stability (A and B) and in vitro clearance (C and D) of human CYP1A2 substrates ramelteon (A and C) and tacrine (B and D) measured in liver microsomes from WT, Cyp1a KO, and hCYP1A1/1A2 mice and human donors (human liver microsomes). Symbols are the measured concentrations of the compounds. A no-NADPH control was also run. Lines are nonlinear regression analysis using the equation for double-exponential (tacrine with Cyp1a KO microsomes) or monoexponential (all other incubations) decay in the software package GraFit 7.0.3. (Erithacus, West Sussex, UK).

The CYP1A2-specific substrate ramelteon exhibited monoexponential decay on incubation with liver microsomes from all vehicle-treated samples and human liver microsomes (Fig. 8A). Ramelteon in vitro clearance in Cyp1a KO and hCYP1A1/1A2 mice and pooled human liver microsomes was ∼83%, ∼190%, and ∼70% relative to WT animals (Fig. 8C). The values of the fraction metabolized by Cyp1a2/CYP1A2 were calculated to be ∼0.17 and 0.56 for WT and hCYP1A1/1A2 mice, respectively (eqs. 15 and 16). Tacrine depletion was monoexponential in all samples except Cyp1a KO microsomes, in which it was preferentially described by the double-exponential decay equation (Fig. 8B). The in vitro clearance of tacrine in pooled human liver microsomes was only ∼12% of that in WT mice, and in microsomes from Cyp1a KO and hCYP1A1/1A2 mice it was ∼32% and 81% of that in WT animals, respectively (Fig. 8D). The calculated Cyp1a2/CYP1A2 fraction metabolized values were ∼0.68 and ∼0.60 for WT and hCYP1A1/1A2 mice, respectively (eqs. 15 and 16).

### Caffeine Pharmacokinetics.

**Fig. 9.**
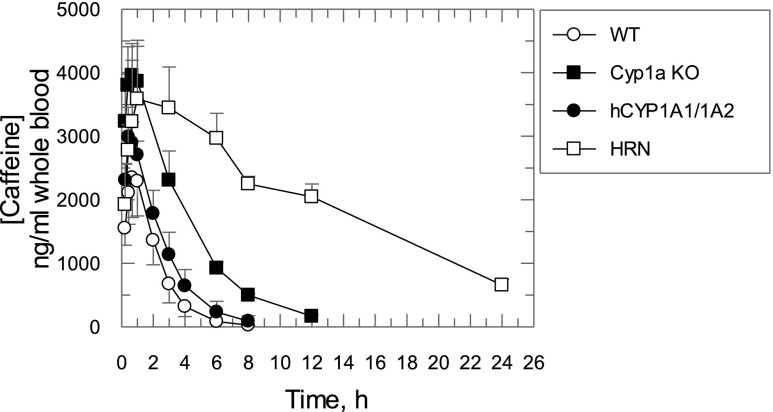
Caffeine pharmacokinetics in WT, Cyp1a KO, hCYP1A1/1A2, and HRN mice. Symbols are caffeine concentrations measured in mouse whole blood. All data are expressed as the mean ± S.D. (*n* = 4 mice per treatment group).

Caffeine is a CYP1A2-specific substrate recommended by the Food and Drug Administration as a sensitive “substrate drug” for in vivo CYP1A2 drug-drug interaction studies. We therefore studied caffeine pharmacokinetics in hCYP1A1/1A2 and Cyp1a KO mice. The pharmacokinetics of caffeine had similar absorption phases in all mouse lines (Fig. 9). There was a small increase (<1.7-fold) in the maximum observed concentration and a minor (<21%) decrease in the apparent volume of distribution in Cyp1a KO, hCYP1A1/1A2, and HRN mice compared with the WT animals (Fig. 9; Table 2). In HRN and Cyp1a KO mice, the elimination half-life was significantly increased (∼8.9- and ∼2.5-fold, respectively) compared with that measured in the WT. In hCYP1A1/1A2 mice, the increase was small (∼1.4-fold) and not statistically significant. The area under the curve (AUC) values were significantly increased (∼10-, ∼3.1-, and ∼1.5-fold compared with WT mice), and apparent clearance values were significantly decreased (to ∼10%, 32%, and 68% of that in WT mice) in HRN, Cyp1a KO, and hCYP1A1/1A2 mice, respectively.

**TABLE 2:** Caffeine pharmacokinetic parameters in hCYP1A1/1A2, Cyp1a KO, WT, and HRN mice Data are the mean ± S.D. (% mean of C57BL/6J ± %S.D.); n = 4.

Mouse Line	C_max_	V/F(obs)	HL	AUCinf(obs)	CL/F(obs)
	*ng/ml*	*ml/kg*	*h*	h*ng/ml	*ml/h/kg*
C57BL/6J	2383 ± 628 (100 ± 26)	1253 ± 494 (100 ± 39)	0.94 ± 0.25 (100 ± 27)	5704 ± 1265 (100 ± 22)	905 ± 170 (100 ± 19)
Cyp1a KO	3991 ± 472** (167 ± 20)	992 ± 188 (79 ± 15)	2.39 ± 0.2*** (253 ± 22)	17651 ± 2073**** (309 ± 36)	286 ± 35*** (32 ± 4)
hCYP1A1/1A2	3082 ± 194 (129 ± 8)	1111 ± 55 (89 ± 4)	1.3 ± 0.33 (138 ± 35)	8397 ± 1797* (147 ± 32)	615 ± 121* (68 ± 13)
HRN	3744 ± 794* (157 ± 33)	1057 ± 82 (84 ± 7)	8.4 ± 0.8**** (891 ± 85)	57,271 ± 2583**** (1004 ± 45)	87 ± 3.8**** (9.7 ± 0.42)

AUCinf(obs), area under the curve from dosing time extrapolated to infinity from the last observed caffeine concentration; CL/F(obs), clearance calculated using AUCinf(obs); C_max_, maximum observed concentration; HL, terminal half-life; V/F(obs), volume of distribution calculated using AUCinf(obs).

^*^ Significantly different from C57BL/6J (unpaired *t* test; two-tailed *P* values; *P < 0.05; **P < 0.01; ***P < 0.001; ****P < 0.0001).

**Fig. 10.**
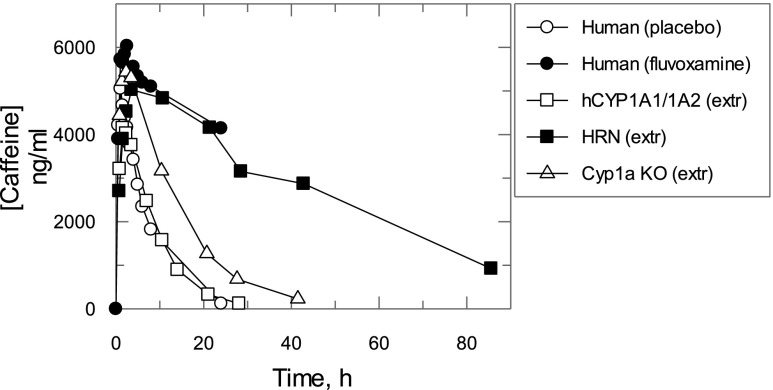
Caffeine pharmacokinetics in hCYP1A1/1A2, Cyp1a KO, and HRN mice extrapolated to humans. Caffeine pharmacokinetics in hCYP1A1/1A2, Cyp1a KO, and HRN mice extrapolated to humans using the complex Dedrick plot approach (see *Materials and Methods* for details). The caffeine concentration time course in placebo- or fluvoxamine-treated healthy subjects (Culm-Merdek et al., 2005) was corrected for mean caffeine concentration in predose. Symbols are the corrected caffeine concentrations measured in human plasma or extrapolated from mouse whole blood. All data are expressed as mean values (*n* = 4 mice per treatment group; *n* = 7 healthy subjects per treatment group).

Caffeine pharmacokinetics in hCYP1A1/1A2, Cyp1a KO, and HRN mice were extrapolated to those in humans by a complex Dedrick plot approach using body weight, clearance, and volume of distribution values in human obtained from Culm-Merdek et al. (2005) and hCYP1A1/1A2 mice to calculate parameters of exponential functions relating clearance and volume of distribution to body weight (Fig. 10; see Supplemental Materials). The data extrapolated from the different mouse lines were compared with caffeine pharmacokinetics observed in healthy human subjects who received placebo or fluvoxamine, a strong CYP1A2 inhibitor (Culm-Merdek et al., 2005). The extrapolated caffeine pharmacokinetics in hCYP1A1/1A2 mice were superimposed with that in humans receiving a placebo, while the extrapolated trace from HRN mice was close to that measured in human subjects after coadministration of caffeine and fluvoxamine. Caffeine pharmacokinetics extrapolated from Cyp1a KO mice demonstrated slower elimination than that in humans with placebo but faster compared with that in healthy subjects after coadministration of fluvoxamine.

## Discussion

We have generated and validated two new mouse models, one where the *Cyp1a* gene cluster has been deleted and one humanized for CYP1A1 and CYP1A2. These models have been used to develop a novel approach to establish the relative roles of CYP1A1 and CYP1A2 in drug disposition. Both CYP1A1 and CYP1A2 are induced as a consequence of the activation of the Ah receptor, and their overlapping substrate specificities have led to considerable interest in developing methods to distinguish their relative contribution to drug oxidation in vitro and in vivo. One approach has been to use selective CYP1A2 inhibitors, such as fluvoxamine/isosafrole (Pastrakuljic et al., 1997; Sy et al., 2001) or furafylline (Stiborova et al., 2002, 2005). Recombinant CYP1A1 and CYP1A2 were used to establish the ratio of activities with isosafrole or fluvoxamine to that without the inhibitor at a single substrate and a number of inhibitor concentrations (Pastrakuljic et al., 1997). Thus, for each inhibitor concentration, there was one ratio for CYP1A1 and one ratio for CYP1A2. The activity in a sample of human liver microsomes was measured with and without the inhibitor, and an equation relating the activities measured in human liver microsomes to the activity ratios in the recombinant enzymes was used to calculate the individual contribution of CYP1A1 and CYP1A2. However, the method has the shortcoming that it uses a single substrate concentration and a limited number of inhibitor concentrations and thus does not use the full magnitude of the kinetic data collected. The latter method (Stiborova et al., 2002, 2005) relies on subtracting activity measured in the presence of furafylline from that without the inhibitor and relating the difference to CYP1A1 concentration measured in human liver microsomes by Western blotting. While the method worked for compounds rapidly metabolized by CYP1A1, e.g., Sudan I, in the case of EROD the approach did not work. Incomplete inhibition of CYP1A2 and contribution from other cytochrome P450s participating in EROD in the presence of furafylline were considered as possible explanations (Stiborova et al., 2005).

The present study exploits the Cyp1a KO model together with quinidine as a specific CYP1A1 inhibitor to define the relative role of CYP1A1/1A2 in drug metabolism, using EROD and MROD as exemplar substrates. Our approach involved the derivation of equations to describe the relationship between reaction rate and substrate and inhibitor concentrations in liver microsomes from hCYP1A1/1A2 mice. This mechanistic approach allows modeling of the CYP1A1, CYP1A2, and non-CYP1A contribution to metabolism of any substrate, at any substrate, inhibitor, or enzyme concentration, and thus can be easily integrated into a PBPK model. Through the use of quinidine as a CYP1A1-specific inhibitor, the CYP1A1 contribution to metabolism of compounds with a slow reaction rate or where CYP1A1 expression is low can be determined.

Hepatic CYP1A1 expression in humans has been reported to be undetectable (McManus et al., 1990; Murray et al., 1993; Edwards et al., 1998, 2003), while others have quantified the enzyme (Drahushuk et al., 1998; Stiborova et al., 2005). The individual contribution of CYP1A1 and CYP1A2 to EROD in a panel of human microsomes has been estimated by selective inhibition of CYP1A2 and CYP1A1 (Pastrakuljic et al., 1997; Sy et al., 2001). In all human samples, the CYP1A1 content was either very low, estimated to be <0.7% of the total hepatic cytochrome P450 (Stiborova et al., 2005), or below the limit of detection, inferring that hepatic CYP1A1 is induced rather than constitutive (Sy et al., 2001). However, due to the very high activity of CYP1A1 toward some compounds (Roberts-Thomson et al., 1993; Kreth et al., 2000; Stiborova et al., 2005, 2015; Li et al., 2007; Lin et al., 2017; MacLeod et al., 2018), it can make a significant contribution to their metabolism even at very low expression levels—for example, aristolochic acid (Stiborova et al., 2015), Sudan I (Stiborova et al., 2005), benzo[a]pyrene (Šulc et al., 2016), granisetron (Nakamura et al., 2005), riociguat (Khaybullina et al., 2014), and erlotinib (Hamilton et al., 2006).

While the measured concentration of CYP1A1 in human liver microsomes is very low [≤3 pmol/mg microsomal protein (Stiborova et al., 2005)], it is inducible in cultured human hepatocytes (Curi-Pedrosa et al., 1994; Liu et al., 2001) and in human liver slices, where an expression level of 25–50 pmol/mg microsomal protein was measured following incubation with TCDD (Drahushuk et al., 1998). The combination of high activity and inducibility makes CYP1A1 a potentially important contributor to variability in toxico/pharmacokinetics of environmental toxicants and/or approved drugs. Indeed, the enzyme can be induced not only by environmental agents but also by prescribed drugs, such as omeprazole, lansoprazole, albendazole, and primaquine (Curi-Pedrosa et al., 1994; Krusekopf et al., 2003; Ueda et al., 2006; Thorn et al., 2012).

In this study, recombinant CYP1A1 metabolized ER with a V_max_ of 3300 pmol/min/mg protein at an enzyme concentration of 71 pmol/mg protein, giving a k_cat_ of 47 minutes^−1^. The concentration of hepatic CYP1A1 in TCDD-treated hCYP1A1/1A2 mice, estimated from the ratio of CYP1A1 EROD V_max_ (2800 pmol/min/mg protein) to k_cat_ (47 minutes^−1^), is 59 pmol/mg microsomal protein. This is in reasonable agreement with the CYP1A1 concentration range of 25–50 pmol/min/mg microsomal protein, obtained in human liver slices incubated with TCDD (Drahushuk et al., 1998), suggesting that any variability in CYP1A1 activity due to induction can be modeled in hCYP1A1/1A2 mice, and its relation to variability in pharmacokinetics of any given drug can be modeled and tested.

CYP1A2 in untreated humanized mice had a much higher activity than Cyp1a2 in the oxidation of ramelteon. Indeed, the contribution of mouse Cyp1a2 to clearance of this substrate was only 17%, whereas in the liver microsomes of hCYP1A1/1A2 mice it was increased to 56%, close to that observed in human liver microsomes in vitro (Obach and Ryder, 2010). Similarly, CYP1A2 exhibited a higher EROD activity than Cyp1a2. The MROD efficacy of both enzymes was similar, whereas tacrine oxidation was faster in liver microsomes from C57BL/6J mice compared with that in the humanized animals. These observations highlight and substantiate the reported species differences in the metabolism of various CYP1A2/Cyp1a2 substrates (Turesky et al., 1998).

**Fig. 11.**
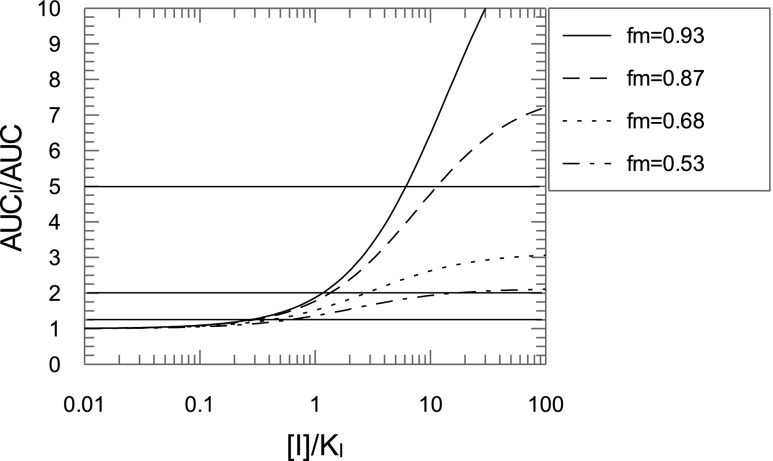
Effect of fraction metabolized on AUC ratios of “substrate drug.” Simulation of AUC ratios of a substrate drug with inhibitor to that without inhibitor as a function of inhibitor concentration for different contributions of the inhibited enzyme [fraction metabolized (f_m_)] to the substrate drug elimination. The enzyme contribution is expressed as part of total clearance. Horizontal lines separate areas with strong (AUC ratio >5-fold), moderate (2 < AUC ratio < 5), weak (1.25 < AUC ratio < 2), and “no effect” (AUC ratio <1.25-fold) inhibition.

The contributions of Cyp1a2 and CYP1A2 to caffeine clearance in WT or hCYP1A1/1A2 were 68% and 53%, respectively (Table 3), suggesting that the mouse enzyme plays a slightly greater role in caffeine disposition. The Cyp1a2 contribution was lower than the 87% established using a Cyp1a2 knockout model (Buters et al., 1996). However, as shown in Table 3, the clearance values for the WT mice in the Buters study were abnormally high, and the clearance measured in the Cyp1a KO mice was the same. In this study, we demonstrated the power of using the Cyp1a KO in conjunction with the humanized mouse to clearly establish the contribution of a particular enzyme in drug elimination. This is important in the study of drug-drug interactions (Fig. 11). At an inhibitor concentration of 10 times K_i_, where the contribution of the enzyme to elimination is 87%, the “substrate drug” AUC will increase approximately 5-fold. This would be considered a strong drug-drug interaction. However, with a 68% enzyme contribution to the drug clearance, the AUC will only increase approximately 2.5-fold, corresponding to a moderate interaction. For a 53% enzyme contribution, the AUC increase will be less than 2-fold and will have a weak effect. The interaction of caffeine with fluvoxamine, a strong CYP1A2 inhibitor, suggested a 93% contribution of CYP1A2 to caffeine metabolism in healthy subjects (Culm-Merdek et al., 2005).

**TABLE 3:** Contribution of CYP1A2 to caffeine systemic clearance calculated from published mouse studies The difference between caffeine systemic clearance in the defined mouse strain and that in the Cyp1a KO mouse line was divided by the value of the systemic clearance in the defined mouse strain and then multiplied by 100% to obtain the contribution of CYP1A2. The clearance value measured in CYP1A2^−/−^ mice was used to calculate CYP1A2 contribution to caffeine systemic clearance in C57BL/6N mice reported by Buters et al. (1996).

Mouse Line	Dose	Route	Clearance	Reference	CYP1A2 Contribution
	*mg/kg*		ml/(h*kg)		*%*
CYP1A2^−/−^	2	i.p.	276	Buters et al., 1996	0
C57BL/6N	2	i.p.	2268	Buters et al., 1996	88
Cyp1a KO	5	PO	286	This study	0
C57BL/6J	5	PO	905	This study	68
hCYP1A1/1A2	5	PO	614	This study	53
C57BL/6J	5	PO	472*^a^*	Li et al., 2017	39
Swiss	20	PO	311*^b^*	Samojlik et al., 2016	8
Swiss	20	i.p.	398*^b^*	Samojlik et al., 2016	28
C57BL/6J	5	PO	726	Scheer et al., 2014	61
CD-COBS	1	i.v.	732	Walton et al., 2001	61
CD-1	20	i.p.	640	Kaplan et al., 1990	55
CD-1	40	i.p.	380	Kaplan et al., 1990	25

PO, per os (oral gavage).

^*a*^ As it was not clear if the AUC reported in the paper was an AUCinf, caffeine clearance was calculated from the C57BL/6J mean pharmacokinetic profile presented in Fig. 2 in the publication (Li et al., 2017).

^*b*^ Clearance was calculated by dividing dose by AUCinf.

When caffeine pharmacokinetics in hCYP1A1/1A2 mice were extrapolated to humans, as described in the *Materials and Methods*, the pharmacokinetic curves were almost identical (Fig. 10). In the case of Cyp1a KO mice, which have no hepatic CYP1A2 activity and are therefore comparable to humans when CYP1A2 is completely inhibited, the extrapolated curve from the null mice suggested notably faster caffeine elimination than that observed in individuals coadministered with the CYP1A2 inhibitor fluvoxamine. However, caffeine elimination extrapolated from HRN mice was superimposable with the fluvoxamine-treated group, suggesting the involvement of P450s other than Cyp1a2 in metabolism. Although this could be considered a confounding factor in the use of the model, it also demonstrates how the model can identify other enzymes involved in drug disposition. A contribution of the murine enzymes to the fraction metabolized is likely to be reduced using the more complex humanized model we have reported recently. In this model, 32 murine P450s from four gene subfamilies have been deleted and substituted for the major human P450s involved in foreign compound metabolism, along with constitutive androstane receptor and pregnane X receptor, the major transcription factors involved in their regulation (Henderson et al., 2019).

The mechanistic approach developed in this study was successfully applied to calculate the individual contribution of human CYP1A1 and CYP1A2 to the metabolism of model compounds ER and MR in liver microsomes from TCDD-treated hCYP1A1/1A2 mice. When applied to a new chemical entity, the method will provide data for the development of a PBPK model, and the predicted interplay between compound concentration and expression of CYP1A1 and CYP1A2 can be tested in vivo using hCYP1A1/1A2 and Cyp1a KO mice. This represents a significant improvement of the currently used in vitro approaches, as it allows the validity of models to be tested in vivo. Humanization for CYP1A1 and CYP1A2, and particularly the use of complex humanized models such as that reported recently (Henderson et al., 2019), will improve the accuracy of extrapolation of preclinical data to humans.

## Abbreviations

AHR: aryl hydrocarbon receptor
AUC: area under the curve
hCYP1A1/1A2: human CYP1A1/1A2
ER: 7-ethoxyresorufin
EROD: 7-ethoxyresorufin *O*-deethylation
HRN: hepatic reductase null
KO: knockout
MR: 7-methoxyresorufin
MROD: 7-methoxyresorufin *O*-demethylation
MS/MS: tandem mass spectrometry
P450: cytochrome P450
PBPK: physiologically based pharmacokinetics
TCDD: 2,3,7,8-tetrachlorodibenzo-*p*-dioxin
WT: wild type
